# Associations between clinically diagnosed medical conditions and dietary supplement use: the US military dietary supplement use study

**DOI:** 10.1017/S1368980023000095

**Published:** 2023-06

**Authors:** Joseph J Knapik, Daniel W Trone, Ryan A Steelman, Emily K Farina, Harris R Lieberman

**Affiliations:** 1 Military Nutrition Division, US Army Research Institute of Environmental Medicine, USARIEM, 10 General Greene Ave, Natick, MA 01760, USA; 2 Deployment Health Research Department, Naval Health Research Center, San Diego, CA, USA; 3 Army Public Health Center, Aberdeen Proving Ground, MD, USA

**Keywords:** Multivitamins/multiminerals, Proteins/amino acids, Herbals, CVD, Mental illness, Osteoarthritis, Cancer

## Abstract

**Objective:**

This study examined associations between multiple dietary supplement (DS) categories and medical conditions diagnosed by health professionals.

**Design:**

Cross-sectional.

**Setting:**

Volunteers completed an online questionnaire on DS use and demographic/lifestyle factors. Medical diagnoses were obtained from a comprehensive military electronic medical surveillance system and grouped into twenty-four clinically diagnosed medical conditions (CDMC).

**Participants:**

A stratified random sample of US service members (SM) from all military services (*n* 26 680).

**Results:**

After adjustment for demographic/lifestyle factors (logistic regression), higher risk was found for 92 % (22/24) of CDMC among individual vitamins/minerals users, 58 % (14/24) of CDMC among herbal users, 50 % (12/24) of CDMC among any DS users and 46 % (11/24) of CDMC among multivitamins/multiminerals (MVM) users. Among protein/amino acid (AA) users, risk was lower in 25 % (6/24) of CDMC. For combination products, risk was higher in 13 % (3/24) of CDMC and lower in 8 % (2/24). The greater the number of CDMC, the higher the prevalence of DS use in most DS categories except proteins/AA where prevalence decreased.

**Conclusions:**

Users in many DS categories had a greater number of CDMC, but protein/AA users had fewer CDMC; results for combination products were mixed. These data indicate those with certain CDMC were also users in some DS categories, especially individual vitamins/minerals, herbals and MVM. Data are consistent with the perception that use of DS enhances health, especially in those with CDMC. Protein/AA and combination product users were more likely to be younger, more physically active men, factors that likely reduced CDMC.

Dietary supplements (DS) are commercially available products consumed as an addition to the usual diet and include vitamins, minerals, amino acids (AA), herbs (botanicals) and a variety of other products^(1)^. More than half of adults in the USA^(2,3)^ and more than 70 % of US military service members (SM)^(4–6)^ use DS. Military personnel and civilians report using DS primarily to enhance health^(6–9)^. Additional reasons SM report for using DS included (in descending order of prevalence) to provide more energy, improve muscle strength, enhance general performance and for weight loss^(6,7)^. Many DS users believe supplements can prevent or treat specific conditions like cancer, heart disease, osteoporosis and depression^(10–13)^, despite limited research supporting these beliefs^(14–18)^.

A number of studies have looked at associations between DS use and medical conditions using US nationally^(19–27)^ or regionally^(11,28–30)^ representative samples. However, these studies have several limitations. First, all studies have depended on self-reports of medical conditions which could be subject to selective recall bias^(31)^. Second, studies have examined a limited number of medical conditions, most notably CVD, cancer, osteoarthritis, hypertension, depression, diabetes and hypercholesterolemia^(11,20,22–30)^. Finally, most studies^(11,20,22,23,25–28,30)^ have focused on vitamins, minerals, and herbal products and have not examined the broader range of DS (as defined by the Dietary Supplement Health and Education Act of 1994)^(1)^ that include proteins, AA, combination products, joint health products and fish oils.

The Armed Forces Health Surveillance Branch of the Defense Health Agency (DHA) captures all clinical encounters between medical care providers and armed forces personnel (Air Force, Army, Marine Corps and Navy) at military medical facilities as well as encounters outside of these facilities paid for by the US Department of Defense. This provides an opportunity to examine clinically diagnosed medical conditions (CDMC) for surveillance or for combining with other datasets if approved by the DHA and institutional review boards. The purpose of the present study was to examine associations between DS use and CDMC. We hypothesised that associations would differ depending on the category of DS used and type of CDMC documented in medical records.

## Methods

This investigation involved a survey of DS use that was combined with electronic medical records of US military SM. It was part of a larger study examining the health effects of dietary supplements^(32,33)^. The investigation was approved by Naval Health Research Center’s institutional review board and SM consented to participate by electronically signing an informed consent document. Investigators adhered to policies and procedures for the protection of human subjects as prescribed by Department of Defense Instruction 3216.01, and the research was conducted in adherence with provisions of 32 Code of Federal Regulations, Part 219.

### Sampling frame and solicitation procedures

Details of the sampling frame, solicitation of SM, subject recruitment flow through the study, statistical power considerations and response bias have been reported elsewhere^(32)^. Briefly, investigators requested from the Defense Manpower Data Center (DMDC) a random sample of 200 000 SM stratified by gender (88 % male and 12 % female) and branch of service (Army 36 %, Air Force 24 %, Marines 15 % and Navy 25 %). The only inclusion criterion was that the individual be an active duty US military SM. Recruitment of SM in this random sample into the study involved a maximum of eight sequential contacts. The prospective participant was first sent an introductory postal letter with a $1 pre-incentive designed to increase the response rate^(34,35)^. The letter also included a description of the survey, a link to a secure website, and a unique number that could be used to access the survey and electronically sign the consent form. As a reminder to those who did not initially complete the survey, a follow-up email message after 10 d and postcard after 3 weeks were sent. If no response was received after sending the postcard, up to five additional email reminders were sent over 8 months, after which contact with the SM ended. All postal and online contacts stated that at any time the SM could decline participation and be removed from the contact list. Recruitment began in December 2018, and no further recruitment was conducted or surveys accepted after August 2019.

### Survey description

The survey was designed to obtain type and frequency of DS use and characterise demographics and lifestyle factors of participants. SM were asked to estimate how frequently they consumed DS in the past 6 months (‘never’, ‘once a month’, ‘once a week’, ‘2–6 times/week’ or ‘daily’). Supplement use questions included ninety-six generic DS (e.g. multivitamins/multiminerals (MVM), individual vitamins and minerals, proteins/AA, and herbal products) and sixty-seven brand-name products. The brand-name products listed included some from previous armed forces DS surveys^(4,6,7,36)^, but the list was updated based on a review of DS sold in the Army, Marine Corps, and Air Force Exchange Systems and General Nutrition Center stores on or near military installations. There were also open text fields on the questionnaire where SM could include supplements not on the provided lists. DS category definitions used in this study are provided in Table 1. To characterise participants, there were questions on demographics (gender, age, formal education, height, weight and military service branch) and lifestyle factors (cigarette smoking, aerobic exercise and resistance exercise).

**Table 1:** Dietary supplement categories in the US military dietary supplement use study

Category	Definition
Dietary supplement	Any substance defined by the Dietary Supplement Health and Education Act^(1)^
Multivitamin/multimineral	DS containing two or more vitamins and/or two or more minerals with no additional supplement ingredients
Protein or amino acid	Amino acid mixtures, protein powders and similar products where the intent is to provide a single or complex protein source
Individual vitamin or mineral	DS that is a single vitamin or mineral supplement, such as vitamin D or Ca
Herbal supplement	DS that includes one or more herbal ingredients with no nutrient or other supplement ingredient. Also includes plant-derived ingredients.
Purported prohormone	Steroidal hormone or herbal substitute for hormones marketed as a DS and included the Supplement Facts panel on the label.
Combination product	DS with mixtures of ingredients from any of the above categories including two or more categories and multiple ingredients. Includes products marketed as weight loss, pre- or post-workout supplements, and muscle/body building products.
Joint health product	Substance that purports to improve the functioning of body joints such as glucosamine (with or without chondroitin) or methylsulfonylmethane
Fish oils	DS that provide *n*-3 fatty acids such as krill oil
Other dietary supplement	Other DS that does not fit into the categories above

DS, dietary supplement.

### Medical data

Once participants were identified by completing the informed consent and survey, the list of participants was sent to the Armed Forces Health Surveillance Branch of the DHA. From the Defense Medical Surveillance System relational database^(37,38)^ the DHA provided investigators all medical encounters of the volunteers for the 6-month period prior to survey completion. Medical encounters in the Defense Medical Surveillance System were recorded as International Classification of Diseases, Clinical Modification, Revision 10 (ICD-10) codes. Encounters included those within military treatment facilities (i.e. Standard Ambulatory Data Record, Standard Inpatient Data Record and Comprehensive Ambulatory/Professional Encounter Record) as well as those outside these facilities (civilian care) and paid for by the US Department of Defense (reimbursable) (i.e. Tricare Encounter Data-Institutional and Tricare Encounter Data-Noninstitutional).

### Statistical analysis

All statistical analyses were conducted using the Statistical Package for the Social Sciences (SPSS) version 26, 2019, SPSS Inc., an International Business Machine (IBM) company. BMI was computed from the questionnaire responses as weight/height^2^ (kg/m^2^). Weekly duration of aerobic and resistance training (min/week) was calculated by multiplying reported weekly exercise frequency (sessions/week) by the reported duration of training (min/session). Supplements that SM placed in the ‘other’ categories were individually examined and responses placed into their appropriate categories. If the listed DS did not fit in a particular category, it remained in the ‘other’ category. Descriptive statistics determined the number and proportion of SM within each demographic and lifestyle characteristic.

ICD-10 codes are a standard system used worldwide by medical health professionals to classify medical conditions diagnosed in patients during clinical visits or hospitalisation. Codes have a leading letter that provides a broad diagnostic category (e.g. infectious disease, circulatory diseases and injury/poisoning), and this is followed by numbers that provide more specific diagnoses within the broader category. ICD-10 code diagnoses of participants were grouped into twenty-four categories shown in Table 2. One series of codes were grouped by their first three ICD-10 alphanumeric codes into nineteen categories representing the major ICD-10 code groups. A separate category included all ICD-10 codes. Four specific code groupings were developed for depression, hypertension, hypercholesterolemia and osteoarthritis to allow specific comparisons with previous literature^(11,19–30)^.

**Table 2:** ICD-10 codes for clinically diagnosed medical conditions in the US military dietary supplement use study (*n* 26 680)

ICD-10 code grouping	CDMC	ICD-10 codes	Participants with CDMC (*n*)
Major code groups	Any CDMC	A00 through Y99	18 775
	Infectious/parasitic diseases	A00 through B99	1870
	Neoplasms	C00 through D49	1069
	Diseases of blood and blood organs	D50 through D89	261
	Endocrine, nutritional, and metabolic diseases	E00 trough E89	2427
	Mental, behavioural diseases	F01 trough F99	3364
	Diseases of nervous system	G00 through G99	3976
	Diseases of eye and adnexa	H00 though H59	4578
	Diseases of ear and mastoid process	H60 through H95	1364
	Diseases of circulatory system	I00 through I99	1395
	Diseases of respiratory system	J00 through J99	4308
	Diseases of digestive system	K00 through K95	1817
	Diseases of skin and subcutaneous areas	L00 through L99	2834
	Diseases of musculoskeletal system	M00 through M99	9688
	Diseases of genitourinary system	N00 through N99	1778
	Pregnancy and childbirth (women only)	O00 through O9A	250
	Congenital abnormalities	Q00 through Q99	285
	Signs, symptoms and abnormal labs	R00 through R99	9132
	Injury/poisoning	S00 through T88	2795
	External causes of morbidity	V00 through Y99	1403
Specific code groups	Depression	F32·0, F32·1, F32·2, F32·3, F32·4, F32·5; F34·8, F32·81, F32·89, F32·9, F33·0, F33·1, F33·2, F33·3, F33·4, F33·40, F33·41, F33·42, F33·8, F33·9; F34·1	763
	Hypercholesterolemia	E78·00, E78·01, E78·1, E78·2, E78·3, E78·4, E78·5, E78·6	663
	Hypertension	I10, I11·0, I11·9, I12·0, 12·9, I13·0, I13·10, I13·11, I13·2, I15·0, I15·1, I15·2, I15·8, I15·9, I16·0, I16·1, I16·9, I67·4, I87·301, I87·302, I87·303, I87·309, I87·311, I87·312, I87·319, I87·321, I87·322, I87·323, I87·329, I87·331, I87·332, I87·333, I87·339, G93·2	958
	Osteoarthritis	M15·0, M15·1, M15·2, M15·3, M15·4, M15·8, M15·9, M16·0, M16·10, M16·11, M16·12, M16·2, M16·30, M16·31, M16·32, M16·4, M16·50, M16·51, M16·52, M16·6, M16·7, M16·9, M17·0, M17·10, M17·11, M17·12, M17·2, M17·30, M17·31, M17·32, M17·4, M17·5, M17·9, M18·0, M18·10, M18·11, M18·12, M18·2, M18·30, M18·31, M18·32, M18·4, M18·50, M18·51, M18·52, M18·9, M19·011, M19·012, M19·019, M19·021, M19·022, M19·029, M19·031, M19·032, M19·039, M19·041, M19·042, M19·049, M19·071, M19·072, M19·079, M19·111, M19·112, M19·119, M19·121, M19·122, M19·129, M19·131, M19·132, M19·139, M19·141, M19·142, M19·149, M19·171, M19·172, M19·179, M19·211, M19·212, M19·219, M19·221, M19·222, M19·229, M19·231, M19·232, M19·239, M19·241, M19·242, M19·249, M19·271, M19·272, M19·279, M19·90, M19·91, M19·92, M19·93	516

ICD-10, International Classification of Diseases, Clinical Modification, Revision 10; CDMC, clinically diagnosed medical condition.

A CDMC was defined as an ICD-10 code within one of the twenty-four code groupings (Table 2). A participant could have an encounter within more than one category but were included only once within a single category. Within each of the twenty-four CDMC, prevalence (as a %) was calculated by DS category for DS users and non-users. Univariable and multivariable logistic regression determined the odds of a CDMC among users and non-users for each DS category. Univariable logistic regression included only the presence or absence of a CDMC (dependent variable) in the DS category. Multivariable logistic regression adjusted the presence or absence of a CDMC (dependent variable) in the DS category for all demographic and lifestyle characteristics (independent variables).

The prevalence of use in each DS category (Table 1) was also examined by the number of CDMC in the nineteen major code groups, exclusive of any CDMC (Table 2). For each participant, the number of CDMC in the nineteen major code groups were determined and placed into one of four groups: 0 (no CDMC), 1–2, 3–4 and ≥5 CDMC. Differences in the prevalence of DS use by the number of CDMC were examined using Chi-square statistics; linear trends across the number of CDMC were examined using the Mantel–Haenszel statistic. The prevalence of any CDMC was also determined by the number of reported DS. DS number was grouped by 0 (non-user), 1–2, 2–4 and ≥5. Univariable and multivariable logistic regression compared the odds of any CDMC according to the number of DS reported.

## Results

From the initial sample frame of 200 000 SM, 73 % (*n* 146 365) were successfully contacted (i.e. no returned postal mail) and of these, 26 680 (18·2 %) signed the informed consent and completed the survey.

Table 3 shows the demographic and lifestyle characteristics of the participants. Participants were primarily men, 30–39 years of age, with an average (sd) of 33 (8) years. Eighty-six per cent had some formal college education or a college degree. Participants varied substantially in time spent participating in weekly exercise, and there was a relatively low proportion of smokers.

**Table 3:** Characteristics of sample in the US military dietary supplement use study by demographic and lifestyle characteristics

Variable	Strata	Sample size *n*	Sample proportion %
Gender	Men	23 037	86·3
	Women	3642	13·7
Age	18–24 years	4660	17·6
	25–29 years	5580	21·0
	30–39 years	11 030	41·6
	≥40 years	5275	19·9
Formal education	Some high school/high school graduate	3879	14·5
	Some college	11 378	42·7
	Bachelor/graduate degree	11 417	42·8
BMI	<25·0 kg/m^2^	7856	30·0
	25·0–29·9 kg/m^2^	13 897	53·1
	≥30·0 kg/m^2^	4425	16·9
Weekly aerobic exercise duration	<91 min/week	7286	27·3
	91–180 min/week	7285	27·3
	181–300 min/week	5869	22·0
	≥301 min/week	6240	23·4
Weekly resistance exercise duration	<45 min/week	7776	29·1
	46–135 min/week	6257	23·5
	136–300 min/week	6581	24·7
	≥301 min/week	6066	22·7
Cigarette smoking	Never smoked	16 706	64·3
	Smoked but quit	4767	18·3
	Smoker	4511	17·4
Service branch	Air force	9788	36·7
	Army	7935	29·7
	Marine corps	3194	12·0
	Navy	5763	21·6

The overall prevalence of CDMC in the 6-month surveillance period was 70·4 % (95 % CI (69·8, 70·9)). Table 4 shows the association between CDMC and use of any DS, MVM, vitamins/minerals, proteins/AA, and combination products. Users of any DS had elevated risk in 13 of 24 (54 %) CDMC; after adjustment for demographic and lifestyle factors, 12 CDMC (50 %) were statistically significant. Among MVM users, risk was elevated in 21 of 24 (88 %); after adjustment, 11 of 24 (46 %) CDMC were statistically significant. Users of individual vitamins/minerals had elevated risk in 23 of 24 (96 %) CDMC; after adjustment, 22 (92 %) CDMC were statistically significant. Users of protein/AA had *lower* risk of a CDMC in 19 of 24 (79 %) CDMC; after adjustment, only 6 (25 %) of these remained statistically significant. Among combination product users, risk of a CDMC was *lower* in 7 of 24 (29 %) CDMC; after adjustment only 3 (13 %) of these remained statistically significant. Also among combination product users, risk of a CDMC was higher in 3 of 24 (13 %) CDMC; after adjustment, 2 (8 %) of these remained statistically significant.

**Table 4:** Clinically diagnosed medical conditions among users and non-users of any DS, MVM, individual vitamins/minerals, proteins/AA and combination products in the US military dietary supplement use study (yellow indicates higher risk among users, green indicates lower risk among users)

	Any DS					MVM					Individual vitamins/mineral				
CMDC	Prev user/non-user %	Unadjusted OR	95 % CI	Adjusted OR	95 % CI*	Prev user/non-user %	Unadjusted OR	95 % CI	Adjusted OR	95 % CI*	Prev user/non-user %	Unadjusted OR	95 % CI	Adjusted OR	95 % CI*
Any CDMC	71·2/68·1	1·16	1·09, 1·23	1·10	1·04, 1·18	72·9/68·4	1·24	1·18, 1·31	1·15	1·08, 1·22	75·6/68·0	1·46	1·38, 1·55	1·33	1·25, 1·41
Infectious/parasitic diseases	7·2/6·5	1·10	0·99, 1·23	1·08	0·96, 1·21	7·3/6·8	1·07	0·97, 1·18	1·04	0·95, 1·15	8·0/6·6	1·24	1·21, 1·37	1·18	1·06, 1·31
Neoplasms	4·1/3·8	1·09	0·95, 1·26	1·07	0·92, 1·24	4·5/3·6	1·27	1·13, 1·44	1·13	0·99, 1·29	5·1/3·5	1·47	1·30, 1·67	1·29	1·13, 1·48
Diseases of blood and blood organs	1·0/0·8	1·32	0·98, 1·78	1·54	1·10, 2·15	1·2/0·8	1·38	1·08, 1·76	1·29	0·99, 1·68	1·6/0·7	2·25	1·76, 2·87	2·15	1·65, 2·80
Endocrine, nutritional diseases	9·6/ 7·7	1·28	1·16, 1·42	1·23	1·10, 1·37	9·9/8·5	1·18	1·09, 1·28	1·05	0·95, 1·15	12/5/7·6	1·18	1·09, 1·28	1·59	1·45, 1·74
Mental, behavioural diseases	13·3/10·6	1·30	1·91, 1·42	1·26	1·15, 1·38	13·2/12·1	1·10	1·02, 1·18	1·06	0·99, 1·15	15·9/11·1	1·10	1·02, 1·18	1·42	1·31, 1·54
Diseases of nervous system	15·4/13·4	1·18	1·09, 1·28	1·16	1·06, 1·26	16·1/14·0	1·18	1·10, 1·26	1·06	0·98, 1·14	18·9/13·1	1·18	1·10, 1·26	1·39	1·29, 1·51
Diseases of eye and adnexa	17·3/16·7	1·05	0·98, 1·13	1·05	0·97, 1·13	18·0/16·5	1·11	1·04, 1·19	1·08	1·01, 1·15	18·8/16·4	1·11	1·05, 1·19	1·12	1·05, 1·22
Diseases of ear and mastoid process	5·2/5·0	1·04	0·92, 1·18	1·03	0·90, 1·17	5·5/4·8	1·16	1·04, 1·29	1·09	0·97, 1·22	5·8/4·8	1·16	1·04, 1·29	1·13	1·00, 1·27
Diseases of circulatory system	5·3/5·1	1·04	0·92, 1·17	1·00	0·88, 1·14	5·8/4·8	1·24	1·11, 1·38	1·11	0·99, 1·25	6·4/4·7	1·24	1·11, 1·38	1·25	1·11, 1·40
Diseases of respiratory system	16·7/14·7	1·16	1·08, 1·25	1·15	1·06, 1·24	17·5/15·1	1·19	1·12, 1·28	1·16	1·07, 1·24	18·9/14·9	1·19	1·12, 1·28	1·26	1·17, 1·35
Diseases of digestive system	7·0/6·2	1·15	1·03, 1·29	1·14	1·01, 1·28	7·4/6·4	1·17	1·06, 1·28	1·09	0·99, 1·20	8·2/6·2	1·17	1·06, 1·28	1·27	1·14, 1·41
Diseases of skin and subcutaneous areas	10·8/10·1	1·08	0·99, 1·18	1·06	0·96, 1·16	11·5/9·9	1·18	1·09, 1·27	1·14	1·05, 1·23	12·5/9·8	1·18	1·09, 1·27	1·23	1·13, 1·34
Diseases of MSK system	37·6/32·6	1·25	1·18, 1·32	1·19	1·11, 1·26	39·1/34·1	1·24	1·18, 1·31	1·14	1·08, 1·20	42·3/33·7	1·24	1·18, 1·31	1·32	1·24, 1·40
Diseases of GU system	7·0/5·8	1·21	1·08, 1·36	1·06	0·94, 1·20	7·7/5·8	1·37	1·24, 1·50	1·14	1·03, 1·27	9·1/5·6	1·37	1·24, 1·50	1·29	1·16, 1·43
Pregnancy, childbirth (women)	7·6/3·9	2·02	1·37, 2·99	2·50	1·64, 3·81	10·3/2·9	3·81	2·77, 5·24	4·14	2·95, 5·81	8·0/5·9	1·38	1·07, 1·78	1·59	1·21, 2·09
Congenital abnormalities	1·1/1·0	1·10	0·84, 1·45	1·03	0·77, 1·36	1·2/0·9	1·31	1·04, 1·66	1·20	0·95, 1·53	1·3/1·0	1·31	1·04, 1·66	1·22	0·95, 1·57
Signs, symptoms, abnormal labs	35·2/31·5	1·18	1·12, 1·25	1·15	1·08, 1·23	36·5/32·4	1·20	1·14, 1·26	1·12	1·06, 1·19	40·3/31·5	1·20	1·14, 1·26	1·34	1·27, 1·42
Injury/poisoning	10·7/9·8	1·10	1·00, 1·20	1·08	0·98, 1·19	11·0/10·1	1·10	1·01, 1·19	1·09	1·00, 1·18	11·9/9·9	1·10	1·01, 1·19	1·20	1·10, 1·31
External causes of morbidity	5·3/5·1	1·05	0·93, 1·19	1·05	0·92, 1·20	5·5/5·0	1·10	0·99, 1·23	1·13	1·01, 1·26	5·9/5·0	1·10	0·99, 1·23	1·19	1·05, 1·34
Depression	3·0/2·4	1·28	1·08, 1·51	1·36	1·13, 1·64	3·0/2·8	1·06	0·92, 1·22	1·05	0·90, 1·23	4·2/2·3	1·85	1·61, 2·13	1·81	1·55, 2·12
Hypercholesterolemia	2·6/2·1	1·25	1·04, 1·51	1·32	1·08, 1·61	2·8/2·2	1·26	1·08, 1·47	1·18	1·00, 1·40	3·2/2·2	1·48	1·27, 1·74	1·42	1·20, 1·68
Hypertension	3·6/3·6	0·99	0·86, 1·14	0·97	0·82, 1·13	4·0/3·3	1·20	1·06, 1·36	1·07	0·93, 1·23	4·2/3·3	1·27	1·12, 1·44	1·14	0·98, 1·31
Osteoarthritis	2·0/1·8	1·11	0·91, 1·36	1·06	0·85, 1·33	2·1/1·8	1·21	1·02, 1·43	1·00	0·83, 1·21	2·6/1·7	1·54	1·29, 1·83	1·34	1·11, 1·63
		Protein/AA							Combination product						
CMDC		Prev user/non-user %	Unadjusted OR		95 % CI	Adjusted OR		95 % CI*	Prev user/non-user %		Unadjusted OR	95 % CI	Adjusted OR		95 % CI*
Any CDMC		68·3/71·9	0·84		0·80, 0·89	0·92		0·86, 0·98	69·6/70·8		0·96	0·91, 1·01	0·99		0·94, 1·06
Infectious/parasitic diseases		7·1/7·0	1·01		0·92, 1·11	1·04		0·93, 1·15	6·9/7·1		0·97	0·88, 1.-06	0·96		0·87, 1·07
Neoplasms		3·3/4·5	0·73		0·64, 0·83	0·95		0·82, 1·10	3·6/4·3		0·84	0·75, 0·96	1·01		0·88, 1·16
Diseases of blood and blood organs		0·9/1·1	0·79		0·62, 1·02	1·17		0·87, 1·56	0·9/1·1		0·82	0·64, 1·05	0·96		0·73, 1·27
Endocrine, nutritional diseases		7·5/10·3	0·71		0·65, 0·78	0·85		0·77, 0·94	8·7/9·4		0·92	0·84, 1·00	0·96		0·87, 1·05
Mental, behavioural diseases		11·6/13·4	0·85		0·79, 0·92	0·93		0·86, 1·01	13·5/11·9		1·15	1·07, 1·23	1·15		1·06, 1·25
Diseases of nervous system		12·3/16·8	0·69		0·65, 0·75	0·80		0·74, 0·87	14·3/15·4		0·92	0·86, 0·99	0·97		0·90, 1·05
Diseases of eye and adnexa		15·8/18·1	0·85		0·79, 0·90	0·94		0·88, 1·02	16·1/18·0		0·88	0·82, 0·93	0·95		0·88, 1·02
Diseases of ear and mastoid process		4·6/5·5	0·82		0·73, 0·92	0·94		0·83, 1·06	4·9/5·3		0·92	0·83, 1·03	1·00		0·88, 1·13
Diseases of circulatory system		3·8/6·2	0·60		0·53, 0·67	0·70		0·61, 0·79	4·4/5·9		0·75	0·67, 0·84	0·79		0·69, 0·89
Diseases of respiratory system		15·2/16·9	0·88		0·82, 0·94	0·93		0·87, 1·01	16·2/16·1		1·00	0·94, 1·07	1·05		0·97, 1·12
Diseases of digestive system		5·9/7·5	0·77		0·70, 0·85	0·89		0·80, 1·00	6·7/6·9		0·97	0·88, 1·06	1·06		0·95, 1·17
Diseases of skin and subcutaneous areas		10·1/11·0	0·91		0·84, 0·98	0·97		0·89, 1·06	10·2/11·0		0·92	0·85, 1·00	0·96		0·88, 1·05
Diseases of MSK system		35·6/36·9	0·95		0·90, 0·99	1·02		0·96, 1·08	37·1/35·7		1·06	1·01, 1·12	1·06		1·01, 1·13
Diseases of GU system		5·8/7·3	0·79		0·72, 0·87	0·95		0·84, 1·07	6·4/6·9		0·93	0·84, 1·02	0·99		0·89, 1·11
Pregnancy, childbirth (women)		4·6/7·9	0·57		0·41, 0·77	0·67		0·48, 0·93	5·0/8·0		0·61	0·46, 0·80	0·69		0·51, 0·94
Congenital abnormalities		1·0/1·1	0·87		0·69, 1·11	0·94		0·71, 1·23	1·0/1·1		0·92	0·73, 1·17	1·02		0·79, 1·33
Signs, symptoms, abnormal labs		32·4/35·6	0·87		0·82, 0·91	0·96		0·90, 1·02	34·2/34·2		1·00	0·95, 1·05	1·04		0·99, 1·10
Injury/poisoning		10·6/10·4	1·02		0·95, 1·11	0·99		0·90, 1·08	10·9/10·2		1·08	1·00, 1·17	1·03		0·95, 1·13
External causes of morbidity		5·3/5·2	1·01		0·91, 1·13	0·98		0·87, 1·11	5·3/5·2		1·03	0·92, 1·14	0·99		0·88, 1·12
Depression		2·4/3·2	0·77		0·66, 0·89	0·97		0·81, 1·15	2·9/2·8		1·03	0·89, 1·18	1·16		0·99, 1·36
Hypercholesterolemia		1·9/2·9	0·63		0·53, 0·74	0·90		0·74, 1·08	2·1/2·8		0·73	0·62, 0·86	0·89		0·75, 1·06
Hypertension		2·4/4·5	0·54		0·47, 0·62	0·61		0·52, 0·72	3·0/4·1		0·73	0·65, 0·84	0·77		0·66, 0·89
Osteoarthritis		1·6/2·2	0·71		0·59, 0·85	0·81		0·66, 1·00	1·9/1·9		0·99	0·83, 1·18	1·10		0·90, 1·33

DS, dietary supplement; MVM, multivitamin/multimineral; AA, amino acid; CDMC, clinically diagnosed medical condition; GU, genitourinary; ICD-10, International Classification of Diseases, Clinical Modification, Revision 10 (refer to Table 2 for ICD-10 code groups); MSK, musculoskeletal; Prev, prevalance.

*Adjusted for gender, age, formal education, BMI, weekly aerobic exercise duration, weekly resistance exercise duration, cigarette smoking and military service branch.

Table 5 shows the association between CDMC and use of prohormones, herbal products, joint health products, fish oils, and other DS. Among prohormone users, risk was *lower* for 1 of 24 (4 %) CDMC, but that CDMC did not remain statistically significant after adjustment. Also among prohormone users, risk was higher in 6 of 24 (25 %) CDMC; after adjustment, risk remained higher in 6 (25 %) CDMC. Users of herbal products had elevated risk in 20 of 24 (83 %) CDMC; after adjustment 14 (58 %) CDMC remained statistically significant. Users of joint health products had elevated risk in 12 of 24 (50 %) CDMC; after adjustment only 4 (17 %) of these remained statistically significant. Also among joint health product users, there was *lower* risk in 1 (4 %) CDMC and after adjustment, 1 (4 %) remained statistically significant. Among fish oil users, risk was elevated in 9 of 24 (38 %) CDMC; after adjustment, 9 (38 %) CDMC were statistically significant. Among users of other DS, risk of a CDMC was higher in 13 of 24 (54 %) CDMC; after adjustment, 10 (42 %) of these remained statistically significant.

**Table 5:** Clinically diagnosed medical conditions among users and non-users of prohormones, herbals, joint health products, fish oils and other DS in the US military dietary supplement use study (yellow indicates higher risk among users, green indicates lower risk among users)

CDMC	Prohormone					Herbal					Joint health product				
	Prev user/non-user %	Unadjusted OR	95 % CI	Adjusted OR	95 % CI*	Prev user/non-user %	Unadjusted OR	95 % CI	Adjusted OR	95 % CI*	Prev user/non-user %	Unadjusted OR	95 % CI	Adjusted OR	95 % CI*
Any CDMC	71·8/70·3	1·08	0·95, 1·22	1·09	0·96, 1·25	75·4/69·1	1·36	1·27, 1·45	1·26	1·17, 1·35	75·8/69·8	1·36	1·22, 1·49	1·21	1·09, 1·33
Infectious/parasitic diseases	5·5/7·1	0·76	0·59, 0·97	0·78	0·60, 1·01	7·6/6·9	1·12	1·00, 1·25	1·07	0·95, 1·21	6·8/7·0	0·97	0·82, 1·14	0·98	0·83, 1·16
Neoplasms	3·2/4·0	0·79	0·58, 1·08	0·92	0·66, 1·23	4·7/3·8	1·23	1·07, 1·43	1·11	0·95, 1·29	5·1/3·9	1·31	1·09, 1·59	1·06	0·87, 1·30
Diseases of blood and blood organs	1·0/1·0	1·02	0·59, 1·79	1·47	0·82, 2·62	1·2/0·9	1·29	0·97, 1·71	1·13	0·83, 1·53	1·0/1·0	0·97	0·64, 1·48	0·92	0·59, 1·44
Endocrine, nutritional diseases	10·9/9·0	1·23	1·03, 1·47	1·18	0·97, 1·43	11·0/8·6	1·31	1·18, 1·44	1·18	1·06, 1·32	10·8/8·9	1·24	1·08, 1·42	1·05	0·91, 1·21
Mental, behavioural diseases	16·1/12·4	1·35	1·16, 1·57	1·35	1·15, 1·58	15·2/12·0	1·32	1·21, 1·43	1·24	1·14, 1·36	13·2/12·5	1·07	0·94, 1·20	1·00	0·88, 1·14
Diseases of nervous system	17·2/14·8	1·19	1·03, 1·39	1·02	0·87, 1·20	17·8/14·2	1·31	1·21, 1·42	1·22	1·12, 1·34	18·4/14·5	1·32	1·19, 1·47	1·10	0·98, 1·23
Diseases of eye & adnexa	15·6/17·2	0·88	0·76, 1·03	0·98	0·83, 1·15	18·0/16·9	1·08	1·00, 1·17	1·06	0·97, 1·15	18·5/17·0	1·11	1·00, 1·23	1·09	0·98, 1·22
Diseases of ear and mastoid process	4·5/5·1	0·86	0·66, 1·13	0·84	0·63, 1·11	5·5/5·0	1·09	0·95, 1·24	1·06	0·92, 1·21	5·5/5·1	1·09	0·91, 1·30	1·00	0·83, 1·21
Diseases of circulatory system	6·1/5·2	1·18	0·94, 1·50	1·10	0·86, 1·41	6·2/5·0	1·25	1·10, 1·42	1·16	1·02, 1·33	5·7/5·2	1·10	0·92, 1·32	0·91	0·75, 1·10
Diseases of respiratory system	15·2/16·2	0·93	0·80, 1·09	1·05	0·90, 1·24	17·1/15·9	1·09	1·01, 1·18	1·04	0·95, 1·13	16·7/16·1	1·05	0·94, 1·17	1·03	0·92, 1·16
Diseases of digestive system	6·5/6·8	0·94	0·75, 1·18	0·98	0·78, 1·25	8·3/6·4	1·32	1·18, 1·47	1·27	1·13, 1·43	7·5/6·7	1·13	0·96, 1·32	1·02	0·86, 1·20
Diseases of skin and subcutaneous areas	10·9/10·6	1·03	0·86, 1·23	1·08	0·90, 1·31	12·6/10·1	1·28	1·16, 1·40	1·21	1·10, 1·33	11·8/10·5	1·14	1·01, 1·30	1·09	0·95, 1·24
Diseases of MSK system	41·6/36·0	1·26	1·13, 1·41	1·15	1·02, 1·30	41·0/35·1	1·28	1·21, 1·36	1·20	1·13, 1·29	46·5/35·3	1·60	1·47, 1·73	1·39	1·27, 1·52
Diseases of GU system	6·2/6·7	0·92	0·73, 1·15	1·37	1·07, 1·75	9·1/6·0	1·56	1·40, 1·74	1·23	1·09, 1·38	7·8/6·5	1·22	1·04, 1·42	1·06	0·90, 1·25
Pregnancy, childbirth (women)	3·3/6·9	0·47	0·06, 3·43	0·70	0·09, 5·38	8·2/6·3	1·34	1·02, 1·75	1·52	1·14, 2·02	4·2/7·2	0·57	0·34, 0·94	0·69	0·41, 1·15
Congenital abnormalities	1·5/1·0	1·48	0·93, 2·34	1·69	1·05, 2·72	1·3/1·0	1·29	0·99, 1·70	1·28	0·97, 1·69	1·5/1·0	1·44	1·02, 2·04	1·37	0·95, 1·97
Signs, symptoms, abnormal labs	35·5/34·2	1·06	0·94, 1·19	1·09	0·96, 1·23	39·1/33·0	1·30	1·22, 1·39	1·22	1·14, 1·30	38·3/33·8	1·22	1·12, 1·32	1·11	1·02, 1·22
Injury/poisoning	13·3/10·3	1·33	1·13, 1·57	1·22	1·02, 1·44	11·5/10·2	1·14	1·03, 1·25	1·11	1·01, 1·23	11·1/10·4	1·08	0·95, 1·23	1·03	0·90, 1·18
External causes of morbidity	6·2/5·2	1·19	0·95, 1·51	1·12	0·88, 1·43	5·3/5·2	1·02	0·89, 1·17	1·01	0·88, 1·16	5·6/5·2	1·09	0·91, 1·30	1·10	0·91, 1·33
Depression	2·8/2·9	0·97	0·69, 1·35	1·22	0·85, 1·73	3·8/2·6	1·46	1·24, 1·70	1·41	1·19, 1·67	3·2/2·8	1·13	0·90, 1·41	1·17	0·92, 1·50
Hypocholesteremia	2·6/2·5	1·06	0·75, 1·50	0·94	0·65, 1·35	3·0/2·3	1·30	1·09, 1·56	1·29	1·06, 1·56	2·9/2·4	1·21	0·95, 1·55	0·94	0·73, 1·23
Hypertension	4·0/3·6	1·12	0·85, 1·47	0·97	0·72, 1·30	4·1/3·5	1·17	1·03, 1·38	1·10	0·93, 1·29	3·6/3·6	1·00	0·81, 1·23	0·77	0·61, 0·98
Osteoarthritis	3·5/1·9	1·87	1·38, 2·52	1·51	1·08, 2·11	2·3/1·8	1·25	1·03, 1·53	1·12	0·90, 1·39	4·1/1·7	2·43	1·96, 2·99	1·89	1·49, 2·38
		Fish oil							Other DS						
CDMC		Prev user/non-user %	Unadjusted OR		95 % CI	Adjusted odds ratio		95 % CI*	Prev user/non-user %		Unadjusted OR	95 % CI	Adjusted OR		95 % CI*
Any CDMC		72·1/69·8	1·12		1·04, 1·19	1·12		1·05, 1·20	76·1/69·9		1·37	1·23, 1·52	1·24		1·12, 1·39
Infectious/parasitic diseases		6·9/7·0	0·97		0·87, 1·09	1·01		0·90, 1·13	7·6/7·0		1·09	0·92, 1·30	1·03		0·87, 1·24
Neoplasms		4·1/4·0	1·04		0·90, 1·20	1·09		0·94, 1·27	4·7/4·0		1·19	0·96, 1·48	1·06		0·85, 1·33
Diseases of blood and blood organs		1·0/1·0	1·00		0·75, 1·34	1·20		0·89, 1·64	1·4/0·9		1·45	0·98, 2·15	1·19		0·78, 1·81
Endocrine, nutritional diseases		10·1/8·8	1·17		1·06, 1·29	1·15		1·04, 1·28	11·5/8·9		1·32	1·15, 1·53	1·20		1·03, 1·40
Mental, behavioural diseases		13·5/12·4	1·11		1·02, 1·20	1·14		1·04, 1·25	18·4/12·1		1·64	1·45, 1·84	1·53		1·35, 1·73
Diseases of nervous system		16·0/14·6	1·11		1·03, 1·20	1·08		0·99, 1·18	18·2/14·6		1·30	1·15, 1·46	1·21		1·06, 1·37
Diseases of eye & adnexa		17·8/17·0	1·05		0·98, 1·14	1·09		1·01, 1·18	18·7/17·0		1·12	1·00, 1·26	1·06		0·94, 1·20
Diseases of ear and mastoid process		5·3/5·0	1·06		0·93, 1·20	1·08		0·95, 1·24	6·2/5·0		1·25	1·03, 1·51	1·15		0·95, 1·41
Diseases of circulatory system		6·1/5·0	1·25		1·11, 1·41	1·23		1·08, 1·40	5·8/5·2		1·14	0·94, 1·38	1·06		0·87, 1·30
Diseases of respiratory system		16·1/16·2	0·99		0·92, 1·07	1·04		0·96, 1·13	19·7/15·8		1·30	1·17, 1·46	1·21		1·07, 1·36
Diseases of digestive system		7·0/6·8	1·03		0·92, 1·15	1·06		0·94, 1·19	9·0/6·6		1·40	1·19, 1·64	1·31		1·11, 1·54
Diseases of skin and subcutaneous areas		10·8/10·6	1·02		0·93, 1·12	1·02		0·92, 1·12	11·6/10·5		1·12	0·97, 1·29	1·06		0·92, 1·23
Diseases of MSK system		39·5/35·4	1·20		1·13, 1·27	1·16		1·09, 1·24	42·6/35·8		1·33	1·22, 1·46	1·25		1·14, 1·39
Diseases of GU system		6·6/6·7	1·00		0·89, 1·12	1·07		0·94, 1·21	9·1/6·5		1·44	1·23, 1·69	1·09		0·92, 1·30
Pregnancy, childbirth (women)		6·1/7·0	0·86		0·62, 1·21	1·09		0·76, 1·55	6·6/6·9		0·95	0·64, 1·40	0·98		0·65, 1·47
Congenital abnormalities		1·2/1·0	1·12		0·85, 1·46	1·21		0·92, 1·60	1·5/1·0		1·42	0·97, 2·08	1·27		0·85, 1·88
Signs, symptoms, abnormal labs		35·5/33·8	1·08		1·02, 1·14	1·10		1·03, 1·17	39·3/33·8		1·27	1·16, 1·39	1·16		1·05, 1·28
Injury/poisoning		10·9/10·3	1·06		0·97, 1·17	1·03		0·94, 1·14	12·3/10·3		1·22	1·06, 1·40	1·22		1·06, 1·40
External causes of morbidity		5·5/5·3	1·07		0·94, 1·21	1·06		0·93, 1·21	5·9/5·2		1·15	0·95, 1·40	1·18		0·97, 1·43
Depression		2·9/2·9	1·01		0·85, 1·19	1·15		0·96, 1·38	4·8/2·7		1·77	1·44, 2·17	1·56		1·24, 1·96
Hypocholesteremia		3·5/2·2	1·59		1·35, 1·87	1·61		1·34, 1·92	2·6/2·5		1·04	0·79, 1·38	1·05		0·78, 1·41
Hypertension		4·4/3·4	1·31		1·14, 1·50	1·29		1·10, 1·51	3·8/3·6		1·05	0·83, 1·32	1·03		0·81, 1·33
Osteoarthritis		2·2/1·8	1·20		0·99, 1·46	1·08		0·88, 1·34	2·1/1·9		1·12	0·82, 1·52	1·05		0·76, 1·46

Abbreviations: 95%CI = 95% confidence interval; CDMC = clinically diagnosed medical conditions (ICD-10 codes); ICD-10 = International Classification of Diseases, Clinical Modification, Revision 10 [refer to Table 2 for ICD-10 code groups]; GU = Genitourinary; MSK = musculoskeletal; Prev = prevalence.

*Adjusted for gender, age, formal education, BMI, weekly aerobic exercise duration, weekly resistance exercise duration, cigarette smoking, and military service branch.

Table 6 shows the prevalence of DS use by the number of CDMC for each DS category. The greater the number of CDMC, the higher the prevalence of use for any DS, MVM, individual vitamins/minerals, herbal products, joint health products, fish oils and other DS. However, as the number of CDMC increased, the prevalence of protein/AA use decreased. For combination products and prohormones, there was no consistent difference in use prevalence as the number of CDMC increased.

**Table 6:** Prevalence of DS use by number of CDMC (nineteen major code groups only) in the US military dietary supplement use study

DS category	Proportion (%) of DS users				*P*-value (Chi-square)	*P*-value (linear trend)
	No CDMC (*n* 7905)	1–2 CDMC (*n* 9772)	3–4 CDMC (*n* 5462)	≥5 CDMC (*n* 3541)		
Any DS	72·0	72·7	76·5	77·8	<0·01	<0·01
MVM	40·8	43·6	48·5	49·6	<0·01	<0·01
Individual vitamin/minerals	25·3	28·8	35·3	41·8	<0·01	<0·01
Proteins/AA	45·2	42·7	41·2	35·8	<0·01	<0·01
Combination products	45·1	43·9	45·4	42·6	0·02	0·11
Prohormones	4·6	4·8	5·2	4·9	0·42	0·11
Herbal products	16·8	19·9	22·3	24·9	<0·01	<0·01
Joint health products	7·7	9·2	10·9	11·5	<0·01	<0·01
Fish oils	21·6	22·9	23·8	24·7	<0·01	<0·01
Other DS	6·2	7·2	8·7	10·8	<0·01	<0·01

DS, dietary supplement; CDMC, clinically diagnosed medical conditions; MVM, multivitamin/multimineral; AA, amino acid; ICD-10, International Classification of Diseases, Clinical Modification, Revision 10 (ICD-10 code groups; refer to Table 2 for specifics).

Table 7 shows prevalence of any CDMC by the number of DS that SM reported consuming. CDMC tended to increase as the number of DS consumed increased, and this trend was more apparent in the adjusted multivariable analysis.

**Table 7:** CDMC by number of DS used in the US military dietary supplement use study

DS reported (*n*)	Sample size (*n*)	CDMC %	95 % CI	Unadjusted OR	95 % CI	Adjusted OR	95 % CI*
None	6571	68·0	66·8, 69·1	1·00		1·00	
1–2	5628	70·1	68·9, 71·3	1·11	1·03, 1·20	1·04	0·96, 1·13
3–4	3784	72·1	70·6, 73·5	1·22	1·11, 1·33	1·13	1·03, 1·24
≥5	10 697	71·4	70·5, 72·2	1·18	1·10, 1·26	1·16	1·08, 1·25

CDMC, clinically diagnosed medical conditions (ICD-10 codes) (refer to Table 2 for ICD-10 codes by CMDC group), DS, dietary supplements; ICD, ICD-10, International Classification of Diseases, Clinical Modification, Revision 10.

* Adjusted for gender, age, formal education, BMI, weekly aerobic exercise duration, weekly resistance exercise duration, cigarette smoking and military service branch.

## Discussion

This study examined the association between CDMC and categories of DS use in a large sample (>26 000) of military SM. Users of many DS categories had a higher prevalence of CDMC than non-users, even after adjustment for demographics and lifestyle factors. A high prevalence of CDMC was especially apparent among users of individual vitamins/minerals, herbal products and any DS (i.e. when use of any DS was analysed). However, protein/AA users had a lower prevalence of CDMC in some categories. The results for combination product users were mixed, but for many CDMC risk was also lower. The prevalence of DS use increased in a linear manner as the number of CDMC increased among users of any DS, MVM, individual vitamins/minerals, herbal products, joint health products, fish oils and other DS. Contrary to this trend, protein/AA use decreased as the number of CDMC increased; for combination product and prohormones, there was little consistent difference in use with an increasing number of CDMC.

Compared with non-users, the lower risk of CDMC among protein/AA and combination product users may be explained in part by the demographic and lifestyle factors of the users. We previously showed in this same cohort^(32)^ and in other samples of SM^(4,6,7)^, that protein/AA and combination products users were more likely to be younger, more physically active men, all factors related to reduced prevalence of medical conditions. Compared with women, men generally use less medical care in military^(39,40)^ and civilian^(41–44)^ populations. These gender-related differences remain after consideration of pregnancy-related conditions and socio-economic characteristics^(39–42)^. Health care use also increases with age^(43,45,46)^ and more physical activity is associated with a reduced likelihood of medical visits^(47–49)^. After controlling for these (and other) factors in multivariate analyses in the current study, the odds for being at greater risk for many types of CDMC was considerably reduced for both protein/AA and combination product users. Nonetheless, a lower risk for some CDMC remained among protein/AA and combination product users, suggesting other factors not examined here might be responsible.

### CVD

Among all non-communicable diseases, CVD is the leading cause of mortality and morbidity worldwide^(50)^, and the American Heart Association estimates that about one-third of American adults have at least one type of CVD^(51)^. In the military, rates of CVD are much lower likely because of SM’s younger age, higher levels of physical activity and lower prevalence of obesity^(52)^. In the current study, SM diagnosed with diseases of the circulatory system reported an overall DS use prevalence (i.e. any DS) similar to those without this CDMC. Nonetheless, risk was higher among users of individual vitamins/minerals, herbals, and fish oils even after adjustment for demographics and lifestyle factors. In agreement, other studies have found that overall DS use was similar among those with self-reported heart disease^(20,24)^. However, when looking at different DS categories, individuals self-reporting many different types of CVDs were more likely to report use of vitamins, minerals and herbal substance in some^(20,22,53)^, but not all^(23,27)^ investigations.

The current study also examined specific CVD code groups for hypertension and hypercholesterolemia, risk factors for CVD, in relation to DS use. There was little difference in overall DS use among those diagnosed with hypertension and those not. While the unadjusted prevalence of MVM, individual vitamins/minerals, herbals and fish oils use was higher among those with hypertension, after adjustment only fish oil use remained higher. In general agreement, several studies examining primarily vitamins, minerals and herbal substances^(20,24,29,53)^ reported little difference in overall DS use (any DS) among those with and without self-reported hypertension. On the other hand, a study of a Southcentral Wisconsin cohort^(30)^ found that self-reported hypertensive individuals were less likely to use MVM.

Overall DS use, as well as use of MVM, individual vitamins/minerals, herbals and fish oils, was higher among those with diagnosed hypercholesterolemia compared with those not using those products. After adjustment, use was still higher in these DS categories among those diagnosed with hypercholesterolemia. Studies have reported that individuals self-reporting hypercholesterolemia^(24)^ or hyperlipidemia^(53)^ had a greater use of any DS, but a study of a Korean cohort^(29)^ found hyperlipidemic individuals were less likely to be users of DS. Differences in defining the specific type of lipids (i.e. total cholesterol, LDL and TAG) may account for a portion of these between-study differences.

Data from the National Health and Nutrition Examination Survey (NHANES) indicated that while both men and women cite improving or maintaining health as the primary reason for using DS, ‘heart health’ ranks high among specific health reasons^(8–10)^. Nonetheless, comprehensive narrative and systematic reviews have shown no clear benefit of vitamins, minerals^(54–56)^ or herbal supplements^(57–59)^ on CVD prevention or treatment. However, it should be noted that some drugs derived from herbals, like aspirin (from willow bark)^(60)^ and reserpine (from *Rauwolfia sepentina*)^(61)^, have become important in CVD treatment. In contrast to vitamins, minerals, and herbs, several systematic reviews have indicated that fish oil supplements (containing the *n*-3 fatty acids, EPA and DHA) may be effective for the secondary prevention of fatal and non-fatal cardiovascular events^(62–64)^. In the current study, SM with diseases of the cardiovascular system, hypertension or hypercholesterolemia were more likely to use fish oils than those without these CDMC even after adjustment for demographics and lifestyle factors.

### Cancer

Cancer is the second leading cause of death in the USA with an annual rate of new cancers of 44·2/1000 person-years in 2013–2017^(51,65)^. In the military, rates of cancer are much lower than in the civilian sector, likely for reasons previously noted^(52)^. In the current study, after adjustment for demographics and lifestyle factors, only individual vitamins/minerals use was associated with higher risk of neoplasms. These data are largely in agreement with other studies that have examined similar associations. The Vitamins and Lifestyle Study involving a regional cohort in western Washington state^(11)^ found that those self-reporting cancer indicated consuming a similar number of DS compared with those not reporting cancer. Analysis of data from the 2015 National Consumer Survey on the Medication Experience and Pharmacist’s Role^(22)^ found that after adjusting for demographics and health status, herbal use was similar among those self-reporting cancer and those not. Data from the Midlife in the US Study^(66)^ showed that those self-reporting cancer were more likely to use DS of any type, but after adjustment for demographics the difference was no longer significant. Secondary analysis of 2017 National Health Interview Survey data^(26)^ indicated that the odds of vitamin/mineral use was 1·39 (95 % CI (1·29, 1·51)) times higher among self-reported cancer survivors than among individuals not reporting cancer.

Individuals may see use of specific vitamins and minerals as a relatively safe way to take a more active role in their treatment^(67,68)^ and certain vitamins and minerals (especially vitamins A, C, D and selenium) were once suggested to have some promise for cancer prevention and treatment, largely because of their antioxidant effects^(69–71)^. Cancer cells produce reactive oxygen species to assist in their growth and survival and antioxidants act to reduce reactive oxygen species by donating an electron and moderating oxidative damage^(72)^. While systematic reviews of observational studies suggest some vitamins may decrease mortality incidence or recurrence for some types of cancers^(73)^, reviews of randomised controlled trials find no clear beneficial or harmful effects of vitamins or minerals on cancer mortality, remission, recurrence, hospitalised days or progression of lesions^(72–76)^.

### Depression

Depression is a leading cause of years lived with disability across the world with about 350 million people suffering from depressive symptoms^(77)^. Among all mental health disorders in the military in 2016–2020, depressive disorders ranked third after adjustment disorders and anxiety^(78)^. The current study found a higher risk of depression among users of any DS, individual vitamins/minerals, herbal products and other DS, compared with non-users. Previously published data on self-reported depression and DS use has not been consistent. Gunter *et al*.^(28)^ found that self-reported depression was associated with greater use of Saint John’s wort. Friedman *et al*.^(24)^ found little difference in overall DS use among those self-reporting depression *v*. those not, while Satia-About *et al*.^(11)^ found that the number of DS used was higher among men self-reporting depression, but not among women.

There have been numerous systematic reviews of the possible efficacy of vitamins, minerals, herbs and fish oils on treatment of depression. Well controlled randomised trials demonstrate that most vitamins, minerals and herbal products examined singly or in combination have little or no effect on depressive symptoms^(79–85)^. However, supplemental folate^(86–88)^ or zinc^(89,90)^ may reduce remission rates and/or depressive symptoms on validated symptom scales when combined with standard antidepressant medications (e.g. serotonin/norepinephrine reuptake inhibitors). Most investigated herbal substances have little effect on depressive symptoms^(91–93)^, but systematic reviews of randomised controlled trials involving Saint John’s wort^(94,95)^, saffron^(96–98)^ and lavender^(99,100)^ suggest some efficacy. Numerous systematic reviews also suggest that supplemental *n*-3 fatty acids may modestly reduce symptoms^(101–105)^.

### Osteoarthritis

Osteoarthritis is a disorder involving deterioration of the articular cartilage and underlying bone and is associated with symptoms of pain and disability^(106)^. Globally, osteoarthritis of the hip and knee ranked as the 11th highest contributor to disability among 291 medical conditions^(107)^, and in the military it was the first or second most common reason for separations from service in 2001 and 2009^(108)^. The current study found that SM with diagnosed osteoarthritis had similar overall use of DS compared with those without osteoarthritis but were more likely to use MVM, individual vitamins/minerals, prohormones, herbals and joint health products; after adjustment, individual vitamins/minerals, prohormones and joint health product use remained higher. In agreement with the present study, Gunter *et al*.^(28)^ found that those with self-reported osteoarthritis were more likely to use joint health products. Rashrash^(22)^ on the other hand found that individuals self-reporting arthritis were more likely to use herbal products, even after adjustment for demographics and other health conditions. Friedman^(24)^ found that those with self-reported arthritis were more likely to use DS of any type, but after adjustment for demographics little difference remained.

Data from systematic reviews of randomised clinical trials indicated that most vitamins, minerals and herbals that have been investigated in osteoarthritic patients do not reduce pain or slow the progression of the disease^(109–114)^, although one review suggested there was moderate quality evidence that extracts of *Boswellia serrata* and avocado-soyabean unsaponifiables may slightly improve pain and function^(112)^. Systematic reviews of randomised clinical trials indicated that oral consumption of the joint health products chondroitin and glucosamine reduced osteoarthritis-related pain but had little or no effect on structural changes (e.g. joint space narrowing and cartilage volume)^(115–119)^.

### Number of clinically diagnosed medical conditions in relation to dietary supplement use

We found a higher number of CDMC associated with a higher number of reported DS use in all DS categories other than proteins/AA, combination products and prohormones. This is consistent with the reported use of DS for presumed ‘health enhancement’^(6–10)^ in that the more medical conditions SM had, the greater use of DS reported in many categories. These data are in agreement with two studies using data from the National Health Interview Study^(19,21)^. Data from the 2012 National Health Interview^(21)^ showed that as number of self-reported chronic conditions increased, there was an increase in use of MVM, individual vitamins, individual minerals and herbal products.

This study was unique in including proteins/AA, combination products and prohormones as a specific category, unlike other studies involving civilians^(11,20–30)^ where there is relatively low number of users of those products. For these DS categories, trends in the association between DS prevalence and the number of CDMC differed considerably from that of other DS categories. Increasing muscle strength is the primary reason protein/AA users report for use of this DS category^(6,7)^, and judicious use of protein/AA combined with resistance training can improve muscle mass and strength over that of resistance training alone^(120)^. The lower use of protein/AA with higher numbers of CDMC may be related to the health of users. As the number of co-morbidities increase, individuals may be less able or have less desire to perform activities to increase strength and concurrently reduce use of proteins/AA.

### Strength and limitations

The current study recruited a very large stratified random sample of SM from all branches of service allowing results to be generalised to the military population. The medical database used in this study contained virtually complete information on diagnosed medical conditions experienced by SM in the surveillance period. The study controlled for multiple demographic and lifestyle factors that could have confounded the associations. Despite these strengths, there were a number of limitations. First, data regarding the DS use and demographic and lifestyle factors were self-reported and share the usual limitations of these types of data, including recall bias, social desirability, errors in self-observation, and inadequate recall^(121,122)^. Second, there were a large number of statistical tests examining relationships between DS use and CDMC. The more effects investigated the greater the chance of making a Type 1 error where the null hypothesis will be incorrectly rejected. Third, the study was cross-sectional, so direct casual inferences cannot be made and the relationships are associative only. Finally, medical conditions were those diagnosed in the 6 months prior to questionnaire completion, and some chronic conditions may have been missed if the SM had not reported to a medical care provider in that period.

## Conclusions

This study had two major findings. First, there were differences in the risk of CDMC depending on the category of DS use. For many categories of DS, association with many types of CDMC was significant, especially among users of individual vitamins/minerals and herbal products. Contrary to this trend, protein/AA users had a lower risk for many types of CDMC and results for combination products was mixed with higher risk for some CDMC and lower risk for others. Proteins/AA and combination products users were predominately male, younger and more physically active, all factors that likely reduced the likelihood of medical conditions and use of the medical system. The second major finding was that the greater the number of CDMC, the higher the DS use prevalence among users of MVM, individual vitamins/minerals, herbal products, joint health products, fish oils and other DS. Again, contrary to this trend, the greater the number of CDMC, the lower the prevalence of protein/AA use, and there was little consistent difference among combination product and prohormone users. This study contributes to the understanding of the association between DS use and medical conditions by examining medical conditions diagnosed by medical care providers, incorporating the full range of medical conditions and by including categories of DS not previously examined in the literature.
